# Proline and COMT Status Affect Visual Connectivity in Children with 22q11.2 Deletion Syndrome

**DOI:** 10.1371/journal.pone.0025882

**Published:** 2011-10-05

**Authors:** Maurice J. C. M. Magnée, Victor A. F. Lamme, Monique G. M. de Sain-van der Velden, Jacob A. S. Vorstman, Chantal Kemner

**Affiliations:** 1 Rudolf Magnus Institute of Neuroscience, Department of Child and Adolescent Psychiatry, University Medical Center Utrecht, Utrecht, The Netherlands; 2 Department of Psychology, University of Amsterdam & The Netherlands Ophthalmic Research Institute, Amsterdam, The Netherlands; 3 Department of Metabolic and Endocrine Diseases and Netherlands Metabolomics Centre, University Medical Center Utrecht, Utrecht, The Netherlands; 4 Department of Developmental Psychology, Utrecht University, Utrecht, The Netherlands; Rikagaku Kenkyūsho Brain Science Institute, Japan

## Abstract

**Background:**

Individuals with the 22q11.2 deletion syndrome (22q11DS) are at increased risk for schizophrenia and Autism Spectrum Disorders (ASDs). Given the prevalence of visual processing deficits in these three disorders, a causal relationship between genes in the deleted region of chromosome 22 and visual processing is likely. Therefore, 22q11DS may represent a unique model to understand the neurobiology of visual processing deficits related with ASD and psychosis.

**Methodology:**

We measured Event-Related Potentials (ERPs) during a texture segregation task in 58 children with 22q11DS and 100 age-matched controls. The C1 component was used to index afferent activity of visual cortex area V1; the texture negativity wave provided a measure for the integrity of recurrent connections in the visual cortical system. *COMT* genotype and plasma proline levels were assessed in 22q11DS individuals.

**Principal Findings:**

Children with 22q11DS showed enhanced feedforward activity starting from 70 ms after visual presentation. ERP activity related to visual feedback activity was reduced in the 22q11DS group, which was seen as less texture negativity around 150 ms post presentation. Within the 22q11DS group we further demonstrated an association between high plasma proline levels and aberrant feedback/feedforward ratios, which was moderated by the *COMT* ^158^ genotype.

**Conclusions:**

These findings confirm the presence of early visual processing deficits in 22q11DS. We discuss these in terms of dysfunctional synaptic plasticity in early visual processing areas, possibly associated with deviant dopaminergic and glutamatergic transmission. As such, our findings may serve as a promising biomarker related to the development of schizophrenia among 22q11DS individuals.

## Introduction

The 22q11.2 deletion syndrome (22q11DS) is a congenital disorder, known to be one of the most common genomic syndromes with an estimated prevalence of approximately 1 in 4000 newborns [1]–[3]. It is characterized by a hemizygous microdeletion on the 22q11.2 segment of chromosome 22 [4], [5]. The 22q11DS is associated with an increased risk for a range of congenital physical malformations including of the palate, heart and face, as well as immune deficiencies [6]. Neurodevelopmental abnormalities include learning disabilities, psychiatric disorders and mild cognitive deficits, with average cognitive function in the range of mild intellectual disability [7]–[9]. Approximately one in four individuals with 22q11DS develop schizophrenia-like psychosis [10], [11]. During childhood a variety of psychiatric disorders are described including attention deficit disorder, obsessive-compulsive disorder and mood disorders [12]–[15]. Also, 20–50% of children with 22q11DS are reported to meet criteria for Autism Spectrum Disorder (ASD) [16]–[18].

Previous studies with 22q11DS individuals mentioned poor performance on perception tasks aimed to study facial memory [19] and facial emotional perception [20]. Andersson et al. [21] demonstrated in their fMRI study among 22q11DS individuals selective anomalies in brain regions associated with face processing, such as the fusiform face area. While these results suggest a high probability of deficits in several aspects of visual perception and processing, they do not indicate from what point in the visual processing stream these anomalies originate. The high prevalence of ASD and psychosis in 22q11DS is of great interest, given that both disorders are also associated with visual processing deficits [22]–[23]. In this respect, the 22q11DS represents a unique model to understand the neurobiological correlates of visual processing deficits related with ASD and psychosis.

The visual system is organized by means of hierarchical streams of processing. Activation first spreads in a feedforward manner from lower to higher visual cortical areas, after which horizontal within-area and feedback connections result in more in-depth processing of the visual stimulus [24]. Feedforward processing results in a representation of the global aspects of a scene at higher cortical levels, called ‘vision at a glance’. Feedback activity, on the other hand, is related with the integration of visual features and is explained as providing detailed information, called ‘vision with scrutiny’. The primary visual cortex, V1, is the starting point of the initial feedforward sweep, which then spreads towards higher regions. Feedback connections are thought to spread from temporal and parietal areas back to lower visual areas. In short, feedforward connections represent feature detection, while more cognitive functions like feature-integration, visual attention and visual awareness rely on feedback connections [25]. Investigation of Event-Related Potentials (ERPs) evoked during a so-called texture segregation task provides us with the possibility of disentangling these different processes.

In the commonly used texture segregation task visual feedforward and feedback processing are dissociated by presenting visual stimuli containing line segments that either make up homogenous fields or checkerboards, and comparing ERPs evoked by these stimuli [26]. Initial feedforward activity is seen in the C1 component, which peaks between 70 and 100 ms after visual presentation and is considered to index afferent activity of visual cortices V1 and V2 [27]. After this feedforward sweep, ERP activity is typically enhanced in response to checkerboards as compared with homogeneous stimuli in time-windows ranging from 100 to 250 ms after stimulus onset. This reflects the isolated neural signal related to recurrent processing between higher visual areas and V1 [25], which is associated with the percept of a figure overlying a background [26]. In the present study, we used the foregoing texture segregation task while recording ERPs from 22q11DS individuals, allowing us to investigate what functional level of visual processing is impaired in this group of individuals, as well as study the integrity of occipital connectivity.

Given the known genetic deletion in these individuals, we were further interested in the link between affected genes and possible visual processing deficits. Previous research in 22q11DS has mostly focused on the involvement of catechol-O-methyl-transferase (*COMT*) and to a lesser extent on proline dehydrogenase (*PRODH*) genes in the neurobiology of 22q11 [28]. The *COMT* gene encodes for the *COMT* enzyme, which plays an important role in the degradation of dopamine, especially in the prefrontal cortex [29]. A common non-synonymous single nucleotide polymorphism (SNP) at *COMT* encodes either valine (val) or methionine (met) at amino acid position 158 of the membrane-bound form of the enzyme. The met allelomorph is unstable at physiological temperature, leading to lower enzyme activity compared to the val variant [30]. Individuals with 22q11DS carry one instead of two copies of the *COMT* gene, as a result of which those individuals with the *COMT* ^met^ genotype may have higher brain dopamine levels than those with the *COMT* ^val^ genotype [28]. The *PRODH* gene, which maps to chromosome 22q11.2, codes for proline dehydrogenase, a mitochondrial enzyme that catalyses the conversion of proline to glutamate. Increased plasma proline levels have been documented in individuals with 22q11DS, which are presumably caused by haploinsufficiency and/or mutations within the *PRODH* gene [31]. Increased plasma proline may significantly alter glutamate neurotransmission, and/or have direct effects on the NMDA receptor [32], [33]. Given that glutamate plays an important role in visual processing, the *PRODH* gene is a plausible candidate susceptibility gene for visual processing deficits in 22q11DS. Further, there is evidence to support a functional association between the *COMT* genotype and proline. This comes from a recent study in which a physiological measure of brain function known as smooth pursuit eye movement was dysfunctional in 22q11DS children with high proline levels, but only when they were carriers of the *COMT* ^met^ allele [34]. Evidence supporting this interaction is available from mice studies in which disrupted brain functioning was found in mice with both high proline levels and low *COMT* activity [35]. In the present study, we tested 1) whether children with 22q11DS display early visual processing deficits and 2) whether these deficits can be related to interactive effects between proline and the *COMT* genotype.

## Materials and Methods

### Ethics Statement

Written informed consent was obtained from each participant before the session, according to the Declaration of Helsinki (2008). The Medical Ethics Committee of the University Medical Center Utrecht approved the study.

### Participants

58 patients with 22q11DS (average age 14.1, ranging 9.1–18.3 years) and 100 typically developing children matched for age (average age 14.6, ranging 7.0–18.9 years) participated in the study. All patients were carriers of a 22q11.2 deletion, as confirmed with positive fluorescence in situ hybridization with adequate probes for the 22q11.2 region carried out in different genetic centers. Four 22q11DS individuals used second-generation antipsychotics (risperidone). All individuals were administered the Wechsler Intelligence Scale for Children, Dutch edition (WISC-III-NL). IQ scores were significantly lower for children with 22q11DS than for children from the control group (Table 1).

**Table 1:** Demographics and medical data.

	Control (n = 100)		22q11DS (n = 58)	
	*N*	Mean (SD)	*n*	Mean (SD)
Age		14.6 (2.7)		14.1 (2.6)
IQ		108 (15)		67 (14)
Male participants	56		23	
Female participants	44		35	
Autistic symptoms	NA		30 (20 males, 10 females)	
ADI social interaction	NA			9.5 (6.3)
ADI Communication	NA			7.3 (4.9)
ADI Stereotypic behavior	NA			2.2 (2.0)
ADI Age of onset	NA			3.8 (1.7)

All parents of children with 22q11DS were administered the Autism Diagnostic Interview-Revised (ADI-R) [36]. 30 out of 58 22q11DS children were diagnosed with an Autism Spectrum Disorder (ASD) and 7 children were diagnosed with a psychotic disorder, 5 of whom with a psychotic disorder NOS, and 2 with paranoid schizophrenia. Diagnoses were based on DSM-IV criteria and were made by a child-psychiatrist. All individuals were free of seizure disorders, neurological diseases, or head trauma. Additionally, before assigning children to the control group, a short questionnaire was used to confirm absence of psychiatric history, and familial history of psychiatric disorders. They were all paid for their participation.

### Proline Measurement and *COMT* ^158^ Genotyping

Plasma proline levels were assessed by automated ion exchange chromatography with post-column ninhydrin derivatization. Plasma amino-acid analyses were performed on a JEOL AminoTac (JEOL AminoTac JLC-500/V, Tokyo, Japan) following AM blood draw. Overnight fast was confirmed in 25 children; in 27 children overnight fasting status was uncertain. One outlier (proline of 580 µM) was identified in the confirmed fasting group, but not removed because abnormally high proline levels can be seen in 22q11DS. Mean proline levels did not differ between the uncertain fasting (278±70 µM) and confirmed fasting (280±110 µM) groups (p = 0.94). Exclusion of the outlier did not alter these results.

*COMT* genotyping was carried out using allele-specific TaqMan probes. Genomic DNA (20 ng) were mixed with the assays and TaqMan® mastermix (Applied Biosystems, Foster City, CA) in a final volume of 5 µl. Four replicates were used for each sample. Samples were treated with the following profile: 95°C for 10 min pre-treatment to activate the Taq Gold and then 40 cycles of 95°C for 15 s and 60°C for 1 min. Data were collected during amplification using the Sequence Detection System software (version 2.2) and a postread run was performed for allelic discrimination.

### Procedure

**Figure 1:**
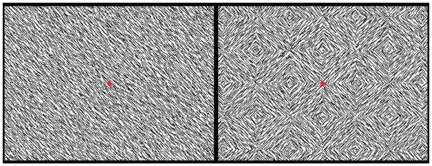
Layout of the task. Examples of the stimuli used: homogeneous (left) and checkerboard stimuli (right) from the texture segregation task. The basic sequence consisted of 16 maps presented in a fixed order; alternating every 550 ms. Randomly during the task Pokemon stimuli were presented in similar size and duration as the line segments. Participants were required to press a button in response to the target Pokemon.

The texture segregation task consisted of full-screen presentations of 900 stimuli on a 21-inch computer screen (42 cm×32 cm) at 1 meter from the participant. The stimuli consisted of homogeneous fields of either 45° or 135° oriented, randomly positioned, line segments (eight different stimuli), or of checkerboards consisting of the same line segments (also eight different stimuli). The homogeneous and checkerboard maps alternated every 550 ms (See Figure 1). The basic sequence consisted of the 16 maps presented in a fixed order (see [37] for details on stimuli and presentation sequence). During presentation a red fixation dot was shown in the middle. Randomly during the task, the stimuli were alternated by Pokemon stimuli, which were presented in similar size and duration as the line segments (in total 39 stimuli, 19 of which were targets). Subjects were required to press a button in response to the target Pokemon.

### Recordings

EEGs were recorded at a sample rate of 2048 Hz from 32 locations using standard Ag/AgCl pin-type active electrodes (BIOSEMI, Amsterdam, the Netherlands) mounted in an elastic cap, referenced to a pair of active electrodes (Common Mode Sense and Driven Right Leg; placed left and right from the Cz electrode) during recording. EEG signals were band-pass filtered (1–30 Hz, and an additional 50 Hz notch filter) off-line and re-referenced to the left mastoid. Horizontal and vertical EOGs were measured for offline correction. The raw data were segmented into epochs from 100 ms pre-target to 400 ms post-target presentation, using Brain Vision Analyzer (Brain Products GmbH, Gilching, Germany). Additionally, ERPs in the first two trials after each Pokemon presentation were not included in the analysis. After EOG correction, epochs with amplitudes exceeding ±100 µV at any channel were automatically rejected. Lowest allowed activity was 3 µV/200 ms, and the maximal allowed voltage step per sampling point was 50 µV. ERPs were baseline corrected using the data for 100 ms prior to target onset.

### Statistical analysis

C1 peaks were scored by an automated procedure at occipital electrodes O1, Oz and O2 as maximal negative peak amplitudes between 60 ms–90 ms. This peak was tested with repeated measures analyses, using as within-subjects variables Stimulus (homogeneous, checkerboard) and Electrode (O1, Oz, O2), as between-subjects factor Group (controls, 22q11DS), and TIQ as a covariate. Further, a difference wave was computed by subtracting the ERPs to homogeneous stimuli from the ERPs to checkerboard (textured) stimuli. The largest negativity in this difference wave (texture segregation negativity) was scored between 130 and 190 ms at the Oz electrode. A univariate analysis of variance with TIQ as a covariate was used to test for texture negativity differences between both groups. The alpha value was set at .05, and only Group main effects or interactions with Group are reported.

*Interaction of COMT^158^ genotype and plasma proline levels on ERP measures.* Our interaction analyses of genotype and proline levels on brain measures were based on recent findings by Vorstman and colleagues [34], who found a clear association between brain measures and proline levels in 22q11DS individuals, which was moderated by the *COMT* ^158^ genotype. For the present analysis of the effect of proline on ERP measures, the 22q11DS group was divided in two subgroups, ‘high proline’ (mean of 344.9 µM, SE 13.9) and ‘low proline’ (mean of 193.6 µM, SE 9.4), based on the group median value of 257 µM. Previous literature indicated that 10^th^, 50^th^ and 90^th^ percentile scores of 16 year old control children were 113, 184, 271 µM [38], which indicates that our ‘high-proline’ group indeed displayed elevated levels of plasma proline. As dependent measure in this analysis we calculated ratio values of feedback/feedforward activity, which was determined by, respectively, texture negativity/C1 amplitudes. This calculation allowed us to working with a single value, reflecting visual feedback activity as a proportion of feedforward activity and thereby reflecting the efficiency of occipital neural transmission. Previous research showed that visual feedback inhibits feedforward transmission, in a direct push-pull mechanism to enhance stimulus contrast [39]. According to this mechanism, feedforward inhibition decreases the figure-ground signal, whereas feedback may increase this. In the present study, ratios of feedback/feedforward activity are assumed to capture this mechanism in a single value. A univariate analysis of variance was used with this ratio as dependent measure, *COMT* ^158^ allele status and high/low proline groups as fixed factors and TIQ as covariate.

## Results

### Behavioral data

No significant differences were found in the total number of responses to target Pokemons between the control group (18.4, SD 1.5) and the 22q11DS group (17.2, SD 2.4) when corrected for TIQ.

### ERP data

#### C1

A Group main effect (*F*(1,152) = 11.6, *p*<.001) showed that children in the 22q11DS group displayed larger negative C1 amplitudes (−3.1 µV, SE .37) than children in the control group (−1.3 µV, SE .24), corrected for TIQ. Similarly for C1 latencies, a Group main effect (*F*(1,152) = 8.3, *p*<.01) showed longer C1 latencies in the 22q11DS group (77 ms, SE 1.3) than in the control group (71 ms, SE .83), also corrected for TIQ. No significant differences on C1 amplitudes and latencies were found between 22q11DS individuals with and without ASD or 22q11DS individuals with and without psychosis.

#### Texture negativity

**Figure 2:**
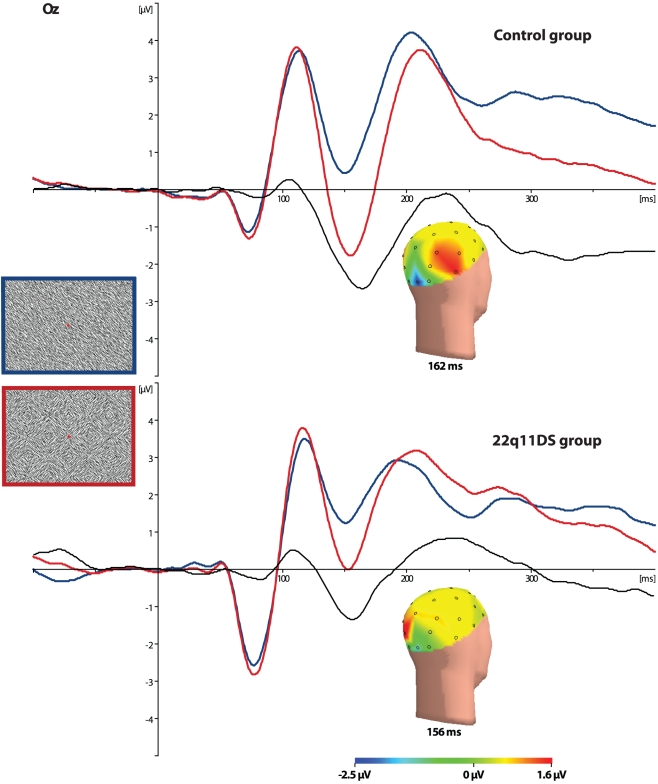
Event Related Potentials. ERPs at Oz electrode representing responses to checkerboards (red lines), homogeneous stimuli (blue lines) and difference waves (black lines). The upper graph represents the control group, the lower graph the 22q11DS group. Voltage maps indicate differential activity between both stimuli at the peak of Oz texture negativity.

A Group main effect (*F*(1,155) = 4.8, *p*<.05) indicated that children with 22q11DS showed smaller texture negativity amplitudes (−1.9 µV, SE .36) than children in the control group (−3.0 µV, SE .24), corrected for TIQ. No significant texture negativity amplitude differences were found between 22q11DS individuals with and without ASD or 22q11DS individuals with and without psychosis. There was no difference in latency between the 22q11DS and control groups (See Figure 2). Ratio values of texture negativity/C1 amplitudes were significantly different between the 22q11DS group (0.65, SE .37) and the control group (1.9, SE .24; *F*(1,146) = 6.1, *p*<.05). Within the 22q11DS group, those individuals diagnosed with ASD showed significantly lower ratio values (0.45, SE .17) than individuals without ASD (1.0, SE .18), (*F*(1,51) = 5.6, *p* = 0.022). No differences were found for ratio values between 22q11DS individuals with or without psychosis.

#### Effects of proline level and COMT^158^ genotype on ERP measures

**Figure 3:**
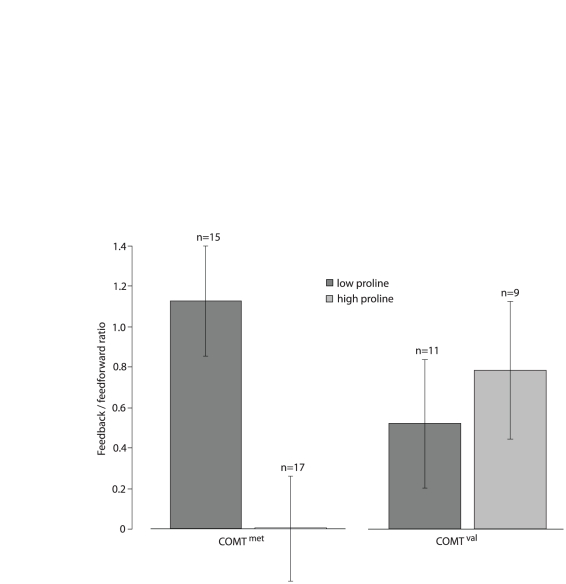
Feedback/feedforward ratio values. The association between proline and feedback/feedforward ratio is moderated by the *COMT* ^158^ genotype. The *COMT* ^met^ subgroup is shown on the left, the *COMT* ^val^ subgroup on the right. Ratio values were calculated by dividing texture negativity amplitudes by C1 amplitudes. The 22q11DS group was further split into ‘high proline’ and ‘low proline’ subgroups, by means of the group median plasma proline value of 257 µM. The control group feedback/feedforward ratio value was 1.9 (SE .24).

We analyzed the effects of proline and *COMT* ^158^ genotype on the ratio between feedback and feedforward ERP activity (texture negativity/C1 amplitude) within the 22q11DS group. Our analysis clearly demonstrated a significant effect of *COMT* ^158^ X proline level on this ratio, when corrected for TIQ (*F*(1,44) = 5.4, *p* = 0.024). A high proline level was associated with a significantly decreased feedback/feedforward ratio in the *COMT* ^met^ group (*F*(1,28) = 4.9, *p* = 0.036), but not in the *COMT* ^val^ group (*F*(1,16) = 0.01, *p* = 0.97, see Figure 3). The control group feedback/feedforward ratio value was 1.9 (SE .24).

## Discussion

In the current study, we measured ERPs to assess visual feedforward and recurrent processing in 58 children with 22q11DS and 100 age-matched controls. Visual feedforward and feedback processing was directly tested by presenting visual stimuli containing either homogeneous line segments or checkerboards, and comparing ERPs evoked by these stimuli. Initial feedforward activity is typically seen in the C1 component, which is considered to index afferent activity of early visual cortex related to feature detection. This is confirmed in the present study as C1 amplitudes did not differ for homogenous or checkerboard stimuli, which are identical with respect to the local features (line segments) that make up the stimuli, and only differ in their perceptual interpretation. Feedback processing was measured by means of the texture negativity wave, which provides a measure for the integrity of recurrent connections in the visual cortical system [25], [27]. Our findings clearly indicate that children with 22q11DS show enhanced feedforward activity starting from 70 ms after visual presentation. After this enhanced feedforward sweep, ERP activity related to visual feedback activity was reduced in the 22q11DS group, which was seen as less texture negativity around 150 ms post presentation. As such, our findings confirm the presence of early visual processing deficits in 22q11DS.

The findings of enhanced feedforward and reduced feedback activity in 22q11DS suggest abnormal transmission between higher and lower visual cortical areas. This interpretation is in agreement with studies on brain connectivity, which reported extensive white matter anomalies in individuals with 22q11DS [40]–[42]. It was suggested that deficits in visual perception but also in social cognition in 22q11DS individuals might be resulting from atypical development and connectivity of occipital brain regions [43]. Van Amelsvoort and colleagues [20] posed that, due to reduced connectivity, individuals with 22q11DS may need to activate occipital brain regions more in order to process visual stimuli. Although the exact mechanisms of this compensatory activity are unknown, our findings are in line with this ‘compensation’ theory, as increased feedforward activity was found in combination with reduced feedback activity.

Within the 22q11DS group we further demonstrated an association between high plasma proline levels and aberrant feedback/feedforward ratios, which was moderated by the *COMT* ^158^ genotype. So far, most genetic studies associated with 22q11DS focused on the *COMT* gene, which plays an important role in the degradation of dopamine [29], and to a lesser extent on the *PRODH* gene, which catalyses the conversion of proline to glutamate [31]. Previous research showed that 22q11DS individuals have increased plasma proline levels [44] that could reflect altered modulation of glutamate production from proline. Evidence supporting proline's role in brain function includes its modulation of glutamatergic neurotransmission in the murine hippocampus in vitro [32], [33] and the presence of high affinity proline transporter molecules in a subset of glutamatergic neurons in the rat brain [45]. Interestingly, we found a negative effect of high proline levels on early visual processing in 22q11DS children with the *COMT* ^met^ genotype. Given the fact that glutamate plays an important role in the neurotransmission within visual pathways, the *PRODH* gene is a plausible candidate susceptibility gene for visual processing deficits in 22q11DS.

An important inference from these findings is that impaired connectivity between visual processing areas in 22q11DS could result from aberrant (functional) synaptic plasticity apart from disconnected (structural) wiring. Glutamate receptors play a central role in excitatory synaptic plasticity in the brain, and are located at multiple levels of the visual system, including retina, lateral geniculate nucleus (LGN), and primary cortex [46]. Misregulation of synaptic communication due to altered glutamate production could therefore lead to impaired functional coupling between early visual processing areas. These deficits can subsequently lead to reported impairments on higher-order processes, such as facial memory [19] and facial emotional perception [20]. Further, modulatory transmitters such as dopamine can change the strength of glutamatergic synapses through various mechanisms [47]. Dopamine can either facilitate or attenuate transmission, depending on the types of receptors [48]. Crucially, in 22q11DS individuals with the *COMT* ^met^ genotype, altered dopamine and glutamate levels might result in aberrant synaptic plasticity in early visual processing areas. However, disconnected structural wiring and impaired functional synaptic plasticity are not necessarily exclusive, and both may contribute to the visual processing deficits that are typical for this disorder. In future studies, it will be important to investigate the exact mechanisms whereby dopamine and glutamate regulate visual processing and how this contributes to psychopathology.

Dysfunctions in synaptic communication are related with various psychiatric conditions, among which are schizophrenia [46] and ASD [49]. Javitt [50] mentioned the importance of NMDA-type glutamate receptors in schizophrenia underlying visual processing deficits, based upon the observation that blocking neurotransmission at NMDA-type glutamate receptors reproduced key symptoms of schizophrenia [51]. Interestingly, there is a growing body of evidence reporting about the impaired reciprocal interactions between dopaminergic and glutamatergic dysfunction in schizophrenia [46]. In this respect, similar pathophysiological mechanisms may underlie visual processing deficits in schizophrenia and 22q11DS. Conversely, atypical visual perception in ASD has been associated with enhanced excitation in early visual brain circuits [52]. This study by Vandenbroucke and colleagues reported strong evidence for intact visual feedback activity in a group of adult, high-functioning ASD individuals, while horizontal connections in lower visual areas were impaired. These findings are in contrast with the present results, indicating that mechanisms underlying visual processing deficits in 22q11DS do not seem to match those underlying ASD. This is surprising, considering the large amount of autistic symptoms in our group of 22q11DS children. One explanation for this discrepancy is that in the current study all children shared at least part of the genetic cause of their ASD (i.e. the 22q11DS), whereas in the Vandenbroucke study ASD in the studied sample could be considered as genetically more heterogeneous. Given the observed age difference between participants in these studies, longitudinal testing may elucidate more in this respect.

Finally, we need to consider the fact that the relation between *COMT* genotype and 22q11DS on cognition seems to depend on the age of the subject [53]. This previous study showed that during childhood, 22q11DS individuals with the *COMT* ^met^ genotype performed better than the *COMT* ^val^ group on test of cognition and IQ, whereas this effect seems to change during adolescence. Our research was conducted with children before and during adolescence, which makes generalization of our effects to an adult group of patients not possible. Also, although we performed statistical corrections for differences in IQ between the control and patient groups, we cannot exclude the possibility that IQ differences might have confounded our results. In a previous study by Jolij and colleagues [54], it was shown that the speed but not the amplitude of recurrent visual processing is influenced by the participants' intelligence. However, the fact that in our study we did find group amplitude instead of latency differences indicates that it is less likely that these should be attributed to differences in IQ.

In conclusion, the current study provides the first ERP data showing early visual processing deficits associated with 22q11DS. Our results show enhanced visual feedforward activity starting from 70 ms after visual presentation, subsequently followed by reduced visual feedback activity, indicating atypical transmission between higher and lower visual cortical areas. Within the 22q11DS group we further demonstrated an association between high plasma proline levels and aberrant feedback/feedforward ratios, which was moderated by the *COMT* ^158^ genotype. These data are discussed in terms of dysfunctional synaptic plasticity in early visual processing areas, possibly associated with deviant dopaminergic and glutamatergic transmission. As such, our findings may serve as a promising biomarker related to the development of schizophrenia among 22q11DS individuals.
